# Solvent-tuned ultrasonic synthesis of 2D coordination polymer nanostructures and flakes

**DOI:** 10.1016/j.ultsonch.2020.105425

**Published:** 2020-12-24

**Authors:** Belén Pepió, Noemí Contreras-Pereda, Salvio Suárez-García, Payam Hayati, Samia Benmansour, Pascal Retailleau, Ali Morsali, Daniel Ruiz-Molina

**Affiliations:** a Catalan Institute of Nanoscience and Nanotechnology (ICN2), CSIC and BIST, Campus UAB, Bellaterra, 08193 Barcelona, Spain; b Instituto de Ciencia Molecular, Parque Científico, Universidad de Valencia, José Beltrán 2, 46980 Paterna (Valencia), Spain; c Institut de Chimie des Substances Naturelles, CNRS UPR 2301, Univ. Paris-Sud, Université Paris-Saclay, 1, av. de la Terrasse, 91198 Gif-sur-Yvette, France; d Chemistry, Faculty of Sciences, Tarbiat Modares University, P.O. Box 14115-4838, Tehran, Islamic Republic of Iran

**Keywords:** 2D MOF, Coordination polymer, Nanomaterials, Delamination, Ultrasound

## Abstract

Herein, a new 2-dimensional coordination polymer based on copper (II), {Cu_2_(L)(DMF)_2_}_n_, where L stands for 1,2,4,5-benzenetetracarboxylate (complex **1**) is synthesized. Interestingly, we demonstrate that both solvent and sonication are relevant in the top-down fabrication of nanostructures. Water molecules are intercalated in suspended crystals of complex **1** modifying not only the coordination sphere of Cu(II) ions but also the final chemical formula and crystalline structure obtaining {[Cu(L)(H_2_O)_3_]·H_2_O}_n_ (complex **2**). On the other hand, ultrasound is required to induce the nanostructuration. Remarkably, different morphologies are obtained using different solvents and interconversion from one morphology to another seems to occur upon solvent exchange. Both complexes **1** and **2**, as well as the corresponding nanostructures, have been fully characterized by different means such as infrared spectroscopy, x-ray diffraction and microscopy.

## Highlights

• Combined ultrasound and solvent assisted synthesis of 2D coordination polymers.
• Role of interstitial solvent molecules in the delamination of 2D-CPs.
• Influence of the sonication time in delamination and nanostructuration processes.
• Morphological and supramolecular transformations in 2D-CPs.

## 1 Introduction

Nowadays much effort is directly or indirectly related to two-dimensional (2D) materials owing to their singular characteristics and their atomic thickness that makes them promising for unimagined properties [1]. For this reason, 2D materials have been proposed for a wide range of applications such as drug delivery [2] imaging agents [3] catalysis [4] energy storage [5] and electronics [6] among others. In fact, much of the success of 2D materials corresponds to graphene due to its outstanding properties such as ultra-high carrier mobility, large specific surface area, optical transparency and high thermal conductivity [7], [8], [9]. Nevertheless, graphene still exhibits some limitations due to the lack of chemical flexibility and its susceptibility to oxidative environments that can hinder its applications. Thus, in the past recent years, different alternatives have been sought to overcome such limitations. One of such families is that of coordination polymers (CPs), whose rational selection of metal ions and directional bonds with organic ligands can be used to control their chemical topology, aiming at finding novel and/or improved optical, electrical and/or magnetic properties [10]. This, together with advances accomplished over the last years towards their miniaturization, offering improved colloidal dispersions, increased surface areas and optimized responses, opened novel prospects for these materials [11].

The fast performance, low cost and environment friendliness of sonochemistry makes it one of the most suitable approaches for the miniaturization of CPs [12] with reproducible morphologies and properties at the micro-/nanoscale [13]. Representative examples are, among others the preparation of: I) Pb(II)-based 2D nanostructures as precursors of lead(II) oxide/bromide [14] II) nickel(II) and cobalt(II) complexes with luminescence, electrochemical and photocatalytic properties [15] and III) CPs that induce the photocatalytic degradation of methylene blue [16] or Congo Red adsorption [17]. Another emerging area where sonochemistry is pushing the limits of miniaturization is that of metal–organic framework nanosheet (MONs). Two-dimensional CPs, commonly known as flakes [19] with enhanced charge transfer between electrolyte and catalysts interface [18], [19] among other applications [20], [21], [22] can be obtained following the top-down liquid-phase exfoliation (LPE) approach. This approach consists on the delamination of the bulk material in the presence of specific solvents, which intercalate between the layers [23], [24] usually assisted with ultrasound (US) [25]. For this, the selection of the solvent is important as suitable polarities will provide stable colloidal suspensions, while some solvents can also decompose or fragment the bulk material, without obtaining layers [26]. Therefore, specific studies have been devoted to determining the role of solvent and US along the delamination process of 2D-CPs [19], [27]. However, there is still a lot of effort to be done given the incipient nature of the field.

**Fig. 1:**
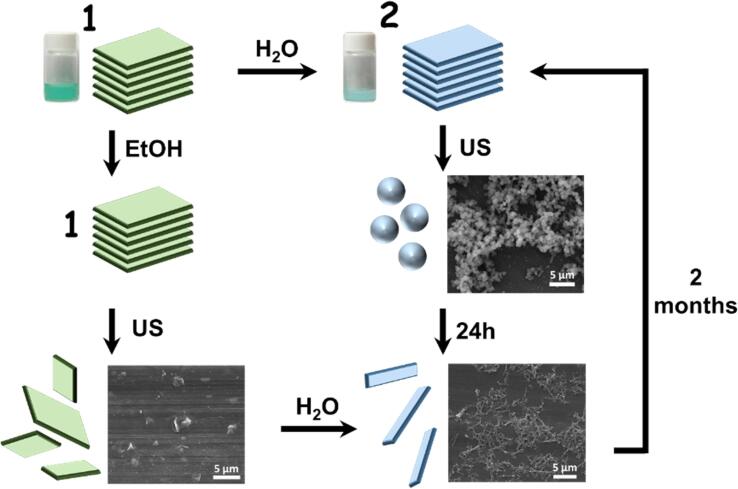
Schematic representation of crystalline phase, color and morphology changes using different solvents and sonication times. Scanning electron microscopy (SEM) and macroscopic images of the sample at various stages are also provided.

Herein we go one step further and demonstrate how the solvent-sonication combination can not only favour the delamination process but also to trigger new reactions and the obtaining of novel chemical nanostructures, otherwise difficult to achieve. A schematic representation of this novel hint is schematically shown in Fig. 1. In brief, crystals of the novel CP {Cu_2_(L)(DMF)_2_}_n_ (**1**), where L stands for 1,2,4,5-benzenetetracarboxylate, have been obtained and later on exfoliated into thin flakes under US in an ethanolic dispersion. Further resuspensions such flakes in water results in a chemical transformation into rod-shaped nanocrystals of complex {[Cu(L)(H_2_O)_3_]·H_2_O}_n_ (**2**). Similar results were obtained if we first disperse bulk crystals of complex **1** in water, which are transformed into crystals of complex **2**, and then we sonicate them. Worth to mention, nanospheres of complex **2** can also be isolated along this process.

## 2 Results and discussion

### 2.1 Synthesis and characterization of complex **1**

**Fig. 2:**
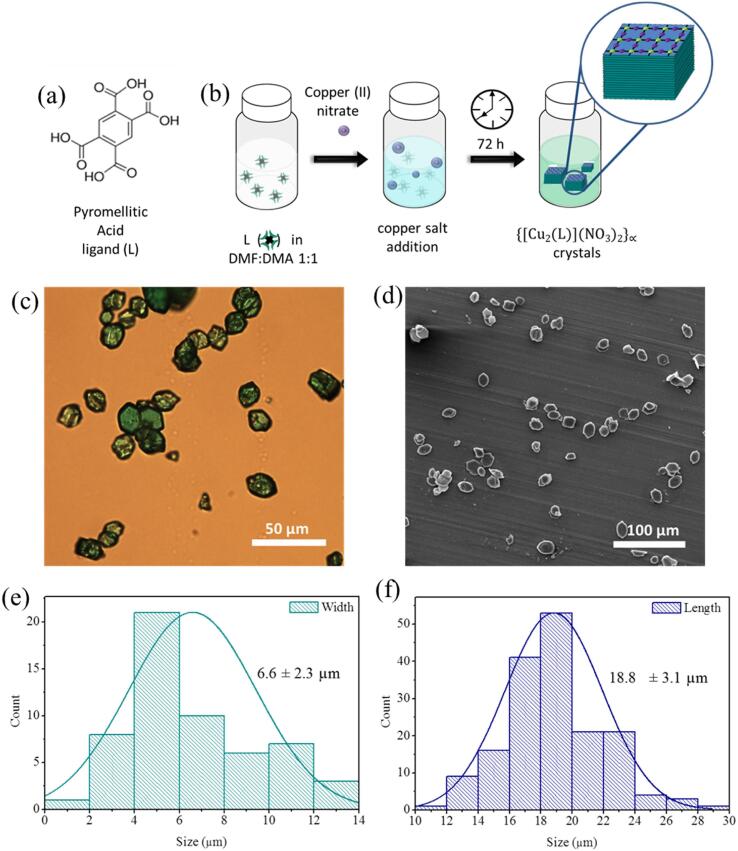
(Top) Synthesis of bulk crystals of complex **1**. a) Chemical structure of the ligand (L) and b) strategy followed for the synthesis of the crystals. (Bottom) Morphology characterization of the crystals: (c) OM image, (d) SEM image, (e) width distribution and (f) length distribution both obtained with the ImageJ software from (d).

Crystals of complex **1** were synthesized by combining the pyromellitic acid as a ligand with four carboxylic groups in 1, 2, 4 and 5 positions and a copper salt (Fig. 2a). The synthesis was performed using a mixture of DMF:DMA (1:1), where the ligand and the metal are soluble (Fig. 2b). Here, the solvent induces the extent deprotonation of the carboxylate ligand (L), consequently facilitating the coordination to Cu(II) ions.

**Fig. 3:**
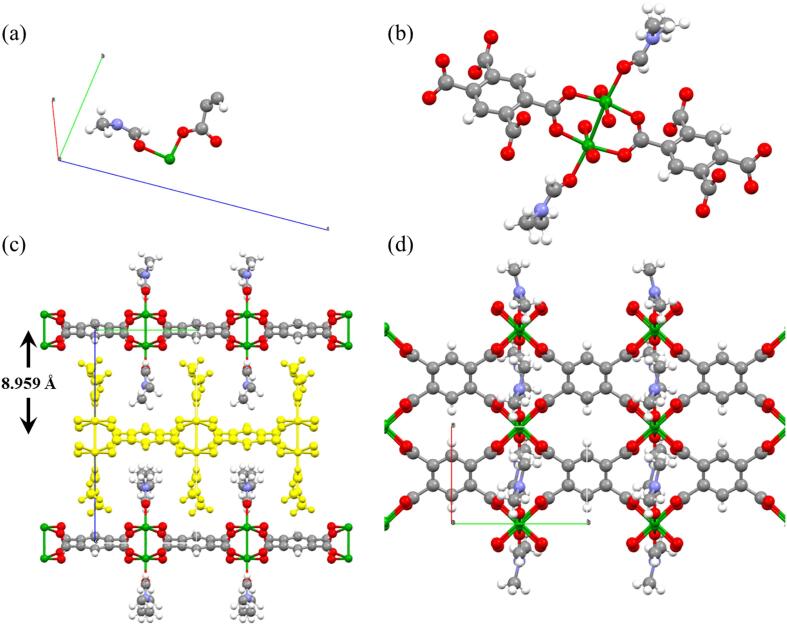
(a) Asymmetric unit of **1**, (b) coordination mode of the metallic centers, (c) layers of **1** from side view, where a single layer is highlighted in yellow, and (d) single layer of **1** from a top view. Color labels: Cu, green; C, grey; O, red; N, blue; H, white. (For interpretation of the references to color in this figure legend, the reader is referred to the web version of this article.)

The morphology and size of crystals **1** were evaluated with both optical microscopy (OM) and scanning electron microscopy (SEM). As seen in Fig. 2c, bulk crystals present a greenish color with an elongated hexagonal shape. SEM images (Fig. 2d) of the bulk crystals were treated with ImageJ software, obtaining a size distribution of 18.8 ± 3.1 μm (N = 178) and a width of 6.6 ± 2.3 μm (N = 56), proving a good monodispersity (Fig. 2e and 2f). Good quality crystals were then selected for single-crystal X-ray diffraction (SXRD) and the results analyzed using Mercury software. Complex **1** is a layered 2D-CP complex with chemical formula {Cu_2_(L)(DMF)_2_}_n_. It crystallizes in the monoclinic space group *I*2*/m*. The lattice parameters of the structure are a = 6.2356(10) (Å), b = 8.6574(12) (Å), c = 17.934(4) (Å), α = 90°, β = 92.365(18)° and γ = 90°. The asymmetric unit of **1** showed in Fig. 3a consists of one Cu(II) ion, one DMF molecule coordinated to the metallic center and one-quarter of the ligand.

As seen in Fig. 3b, the metallic center of the compound consists of two coordinated Cu(II) ions forming a dimer in a paddlewheel structure. Cu(II) ions are coordinated to the carboxylate groups of the ligand in the equatorial positions in a tetradentate mode, each oxygen from a carboxylate being coordinated to one Cu(II) of the dimer. In order to complete the paddlewheel geometry, DMF molecules are coordinated in the remaining axial positions. Regarding the crystalline structure, Fig. 3c-d show both the layers from the top and side view. The structure showed in Fig. 3d undergoes extended coordination leading to the formation of a 2D-sheet CP. These layers pack themselves through supramolecular interactions, mainly van der Waals interactions to form the bulk layered crystal, as seen in Fig. 3c, where a single layer is highlighted. Layers remain along (0 0 1) planes with a distance between them of 8.959 Å. More crystallographic data can be found in Table S1 and S2 (Supporting Information). Besides, powder X-ray diffraction (PXRD), Fourier Transformed Infrared (FT-IR) and energy dispersive x-ray (EDX) spectroscopies were performed in order to corroborate the powder crystalline pattern, the formation of the CP and the presence of C, N, O and Cu in the bulk crystals of **1** (Figure S1, Supporting Information).

### 2.2 Solvent effect

**Fig. 4:**
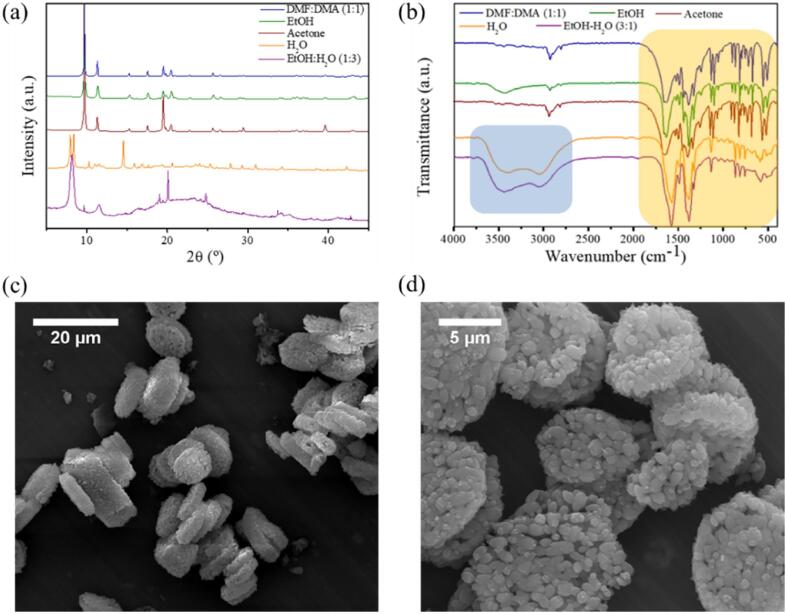
(Top) Characterization of 2D-CP crystals in different solvents. (a) PXRD and (b) FT-IR spectra. (Bottom) SEM images of (c) bulk 2D-CP immersed in water and (d) bulk 2D-CP resuspended in DMF:DMA (1:1) after being 24 h in EtOH-H_2_O (3:1).

Bulk crystals of complex **1** were resuspended for 24 h in ethanol (EtOH), acetone, water and a mixture of EtOH:H_2_O (1:3) and analyzed by PXRD and FT-IR spectroscopy (see Fig. 4a-b).

As can be seen there, results for EtOH and acetone reproduced the patterns for the as-synthesized crystals. However, significant differences were found for those suspended in water and the EtOH:H_2_O (1:3) mixture. In this last case, FT-IR analysis revealed the presence of O–H bonds (characteristic peak within the range of 3600 – 2700 cm^−1^ highlighted in blue) and a displacement of the peaks to lower wavenumbers (area highlighted in yellow), tentatively attributed to an exchange of solvent molecules coordinated to Cu ions. In fact, PXRD evidenced changes in the crystalline phase after 24 h along with irreversible fragmentation into smaller granular crystals, as confirmed by SEM (see Fig. 4c-d). On top of that, a simple qualitative observation already revealed that the crystals become bluish immediately after suspension in water or EtOH:H_2_O (1:3) mixture. Finally, the irreversibility of the interconversion was tested by immersing back the crystals in DMF up to two months; no significant changes were detected in the corresponding FT-IR and PXRD spectra (see Figure S2).

### 2.3 Synthesis of complex **2**

**Fig. 5:**
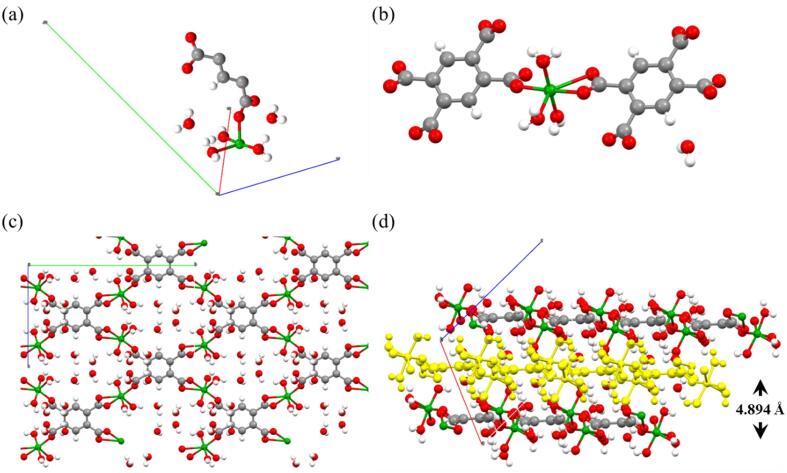
(a) Asymmetric unit of **2**; (b) Coordination mode of the Cu(II) metallic centers to L; (c) Top view of a single layer of **2** and (d) side view of packed layers, where a single layer is highlighted in yellow. Color labels: Cu, green; C, grey; O, red; H, white. (For interpretation of the references to color in this figure legend, the reader is referred to the web version of this article.)

To fully determine the influence of water in complex **1,** crystals were then immersed for a longer period of one month. Interestingly, the seeds shown in Fig. 4 grew more to form hexagonal bluish crystals, suitable for SXRD (Figures S3, S4 and S5, Supporting Information). Such crystals corresponded to the new complex **2**, which was also a 2D-CP complex with a layered structure of chemical formula {[Cu(L)(H_2_O)_3_]·H_2_O}_n_ that crystallizes in the monoclinic space group *I*2*/a*. The lattice parameters of the structure are a = 9.4869(3) Å, b = 18.0136(6) Å, c = 12.0060(4) Å, α = 90°, β = 112.734(4)° and γ = 90°. Fig. 5a shows the asymmetric unit of the complex made of a metallic Cu(II) center coordinated to three water molecules and a half of the ligand. There are also present two interstitial water molecules. Furthermore, the metallic center could be found in the structure with a deformed octahedral coordination sphere (Fig. 5b). The Cu(II) center is coordinated to two L through the carboxylate groups both in a monodentate and bidentate mode in the equatorial positions. Thus, to complete the coordination sphere, the remaining equatorial position is occupied by a molecule of water as well as the axial positions and expanded through a plane forming a 2D-sheet of **2** (Fig. 5c). The layers pack themselves both through van der Waals interactions and hydrogen bonds, allowing the growth of the bulk layered crystal. Hydrogen bonds are formed between adjacent layers in a zig-zag way conferring higher stability to the bulk form of **2**. The aforementioned layers remain within (10–1) planes, with a distance between them of 4.894 Å (Fig. 5d). After a detailed search, we found that complex **2** was already described in the literature following a direct synthetic approach [28].

### 2.4 Tip-assisted sonication of complex **1** in water

**Fig. 6:**
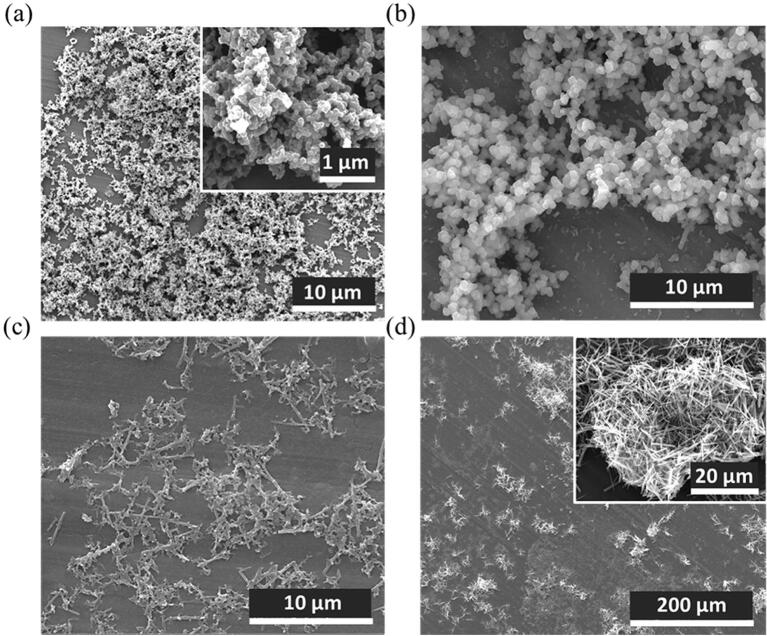
SEM images of the colloidal suspensions sonicated for 2 h (a) after sonication, (b) and (c) after 24 h and (d) precipitated obtained in the colloidal suspensions after 24 h.

In the previous sections 2.2 and 2.3, we have shown how the dispersion of crystals of complex **1** in water induced a chemical transformation over time (already detectable at 24 h) that ends in the formation of crystals of considerable dimensions with time. Next step was the combination of water effect with US. For this, US were applied to an aqueous dispersion of **1** with a 550 W powered microtip in pulses of 2 min followed by 30 s of rest between steps at 25% tip amplitude energy for 2 h (this was the optimized time in terms of polydispersion with no significant changes in morphology, see Table S3 Supporting Information). US microtip provides large powered US generated at a small source (the end of the tip) resulting in large power intensities per surface area of ~ 1700 W·cm^−2^ even at 25% of its amplitude. Generating this high intensity in-situ the suspension induces large-yielded nanostructuration. It is also worth to mention that large US power density of ~ 34000 W·L^-1^ average is obtained in this system as small volumes are used for the suspensions despite a decreasing gradient of US power density is expected upon moving away from the tip. To ensure the homogenization of the sample, the crystals were maintained under magnetic stirring and with a water–ice bath to avoid thermal decomposition. Dynamic light scattering (DLS) measurements revealed the formation of colloidal suspensions with average dimensions of 62.2 ± 7.2 nm and a polydispersity index (PDI) value of 0.331 ± 0.017 revealing the importance of large US energies to obtain nanometered structures of CPs. The relatively high PDI value was attributed to the inhomogeneous sonication through the solution from the tip as aforementioned. Similar results were obtained using now a sonication bath (where US power is applied more homogenously over all the crystal suspension) only improved after exposition for longer periods of time (see Table S4 and Figure S6, Supporting Information) due to the low power intensity and density of this set-up. In a US bath, US are generated along the large surface of the bath resulting in intensities 0.1 W·cm^−2^ with the equipment used in this work. Further, despite having a more uniform gradient, US propagate over the voluminous water bath leading to weak power densities (33 W·L^-1^).To further improve the PDI, the remaining non-exfoliated material can be removed by additional centrifuge optimization after sonication with the microtip. Specifically, colloidal suspensions sonicated for 2 h were centrifuged at 13300 rpm and 4 °C at different times of 2 min, 5 min, 7 min, 10 min, 15 min and 20 min. SEM images (see Figure S7, Supporting Information) pointed out to 10 min as an optimized centrifugation time. As shown in Fig. 6a-b, sonication for 2 h and use of the optimized centrifugation conditions, resulted in nanospheres of mean size 79.8 ± 20.5 nm (N = 106) (see Figure S8, Supporting Information for size statistics). Interestingly, the colloidal suspension evolves after 24 h to form larger nanospheres that finally tended to form less stable rods that ultimately precipitate (Fig. 6c-d).

**Fig. 7:**
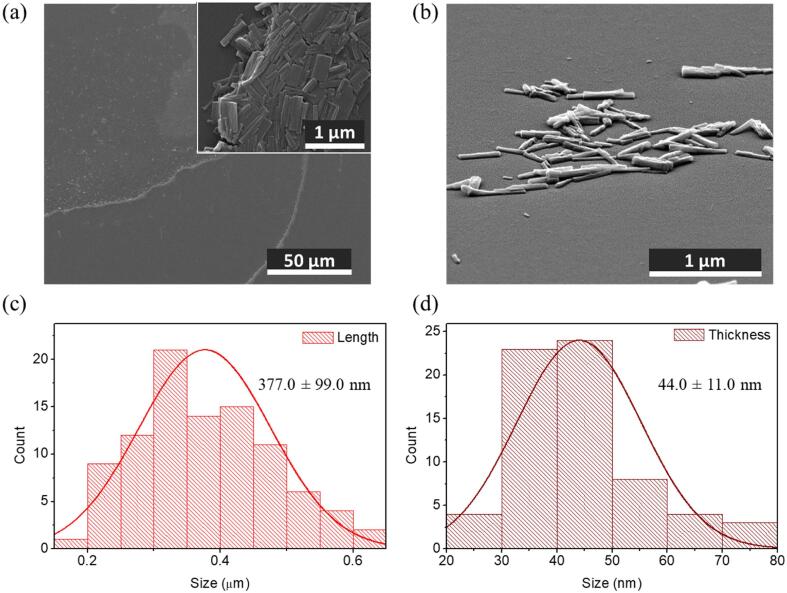
SEM images of (a) top and (b) tilted view of the nanostructures deposited onto a surface by spin coating (5000 rpm, 30 s). Size distribution for (c) length and (d) thickness of the obtained nanostructures.

Top view and tilted SEM images of the nanorods shown in Fig. 7 revealed a mean length of 377.0 ± 99.0 nm (N = 95) and a mean thickness of 44.0 ± 11.0 nm (N = 66). Regarding FT-IR analysis (Figure S9a, Supporting Information) most of the peaks at lower wavenumber were maintained, indicating chemical stability during the US process. On the other hand, comparing the peaks of the Grazing angle X-Ray Diffraction (GXRD) in Figure S9b, the pattern from the nanostructures was similar to that of complex **2**, with a main diffraction peak attributed to the plane along the 2D layers of **2** (2θ = 9.8°). In fact, immersion in water for longer periods of time (three months) of the precipitate obtained after exfoliation leads to the formation of isostructural hexagonal crystals (Figure S10, Supporting Information) complex **2**, as confirmed by SXRD.

### 2.5 Tip-assisted sonication of complex **1** in EtOH

**Fig. 8:**
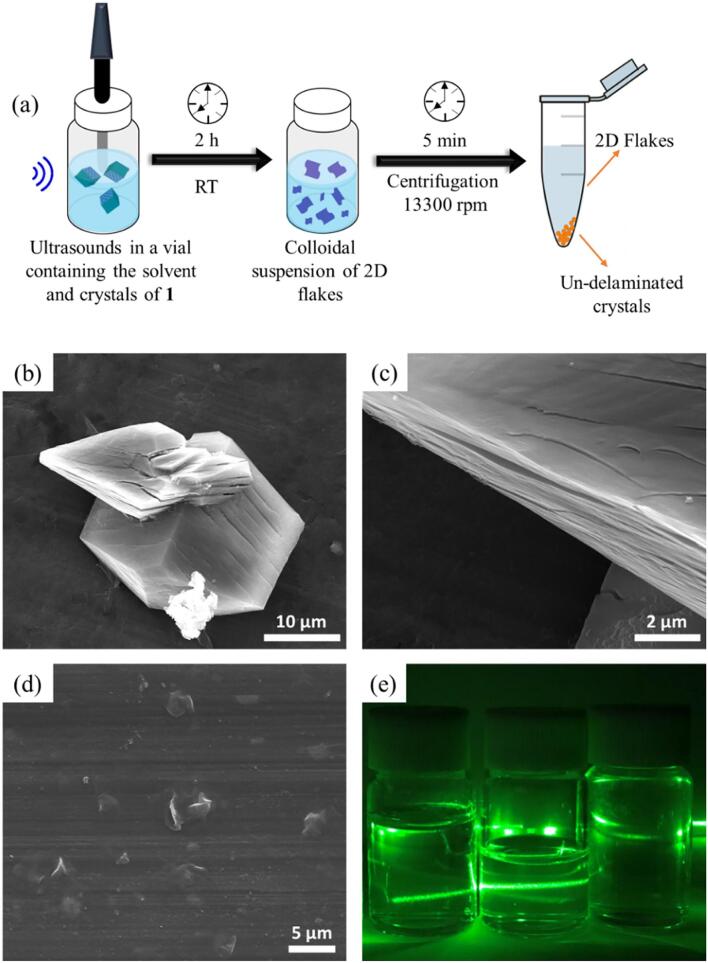
(a) Scheme of the LPE process applied for the delamination of bulk 2D-CP crystals using US; (b) and (c) SEM images of the layered 2D-CP bulk crystals exposed to EtOH for 24 h, where a preliminary exfoliation can be appreciated; (d) SEM images of flakes of complex **1** upon sonication in EtOH; (e) Tyndall effect observed in colloidal suspensions of flakes obtained with the tip in different solvents compared to water solvent without flakes (from left to right: EtOH, water suspensions and water solvent).

Crystals of complex **1** dispersed in EtOH for 24 h already showed an incipient process of delamination as can be seen in Fig. 8 b-c. Though, in order to optimize the exfoliation process with the ultimate goal of reaching very-thin flakes, an US microtip was immersed in the colloidal dispersion for 2 h as large energetic source to increase the exfoliation rate (Fig. 8a). Crystal dispersions were maintained under magnetic stirring for the homogenization of the sample and kept at room temperature using a water–ice bath to avoid thermal decomposition. US were applied in pulses of 2 min, followed by 30 s of rest between steps at 25% tip amplitude energy as in section 2.4. SEM images showed the obtaining of flakes (Fig. 8d) with an average size of 101.5 ± 4.5 nm with a PDI of 0.254 ± 0.011 determined by DLS measurements. Once more, sonication with a US bath resulted in similar flakes and PDI values only after long sonication times to enlarge the US energy provided to the crystals (Table S4, Supporting Information). Finally, the flakes showed excellent colloidal stability in EtOH (Fig. 8e) up to one month after the exfoliation (157 ± 74 nm and PDI of 0.232 ± 0.001).

**Fig. 9:**
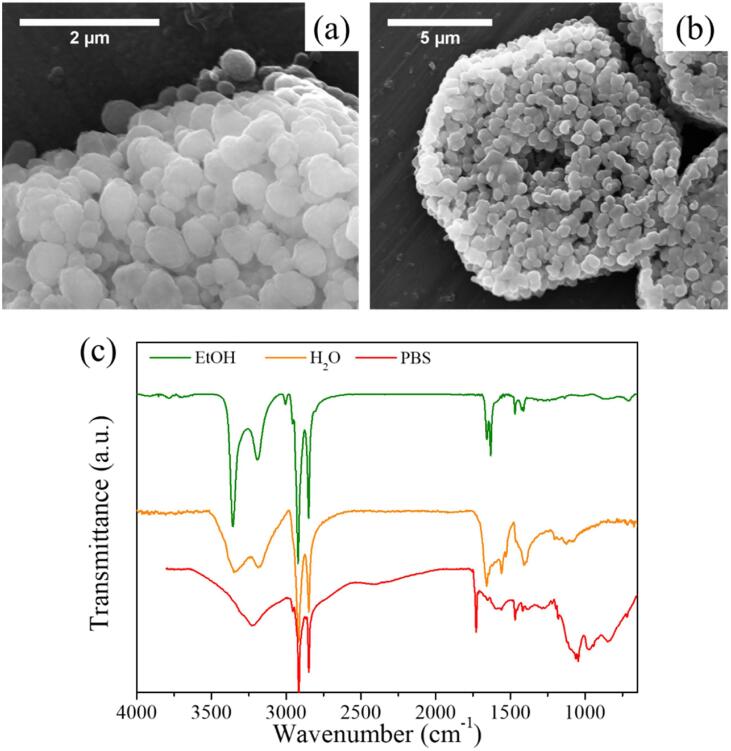
(a) and (b) SEM images of flakes previously exfoliated resuspended in water after EtOH evaporation. (c) FT-IR spectra of flakes resuspended in different solvents.

Finally, the flakes shown in 8b were re-suspended in water and PBS (in order to mimic physiological conditions). For this, a combination of equivalent volumes of the flake dispersion in EtOH and water/PBS was rotaevaporated until EtOH was removed. DLS measurements of the resulting dispersions showed values of 153.7 ± 4.0 nm (PDI: 0.366 ± 0.029) and 122.9 ± 28.8 nm (PDI: 0.365 ± 0.043) for water and PBS, respectively. Morphology was only analyzed with SEM for flakes resuspended in water (Fig. 9) due to the presence of saline microcrystals in PBS. The change of morphology was evident as the flakes adopt a flattened sphere shape (Fig. 9a), similar to the nanospheres obtained after applying US to crystals of **1** in water seen in section 2.4. After five days in water, flakes reorganize in a hexagonal way (Fig. 9b) reminding of the shape of crystals of **2** after several months. SEM image presented in Figure S11 corresponds to the resuspension before EtOH evaporation. It shows a mixture of the sheet-shaped flakes typically found in EtOH and flattened sphere-shaped flakes when resuspending in water. Thus, change in morphology was attributed to the presence of water.

FT-IR spectra were obtained for both resuspensions in water and PBS. Fig. 9c compares the FT-IR spectrum from the flakes exfoliated in EtOH and the resuspended ones. It seems that chemical structure was not retained when redispersing in water and PBS as O–H bonds (3000–3500 cm^−1^) in the structure appear to arise as happened when immersing the bulk crystals in water. Changes at lower wavenumbers were also appreciated, although lack of resolution of the peaks did not allow us to fully note the shift in the peaks, which could be an indicator of novel coordination sphere.

## 3 Conclusions

A novel 2D Cu(II) CP of chemical formula {Cu_2_(L)(DMF)_2_}_n_ (**1**) has been synthesized and characterized. The structure obtained by SXRD revealed coordination of copper ions in a paddlewheel geometry in 2D layers, including coordinated DMF molecules. Stability of the bulk crystals in different solvents was studied. Water caused irreversible crystalline changes into the complex to form complex **2**, a 2D-CP with a layered structure of chemical formula {[Cu(L)(H_2_O)_3_]·H_2_O}_n_ previously reported in the literature [28] where coordinated DMF molecules got replaced by water coordinated to the copper metallic center. Combination of water with US resulting in the formation of nanoparticles after 2 h that evolve with time into rods that precipitate after 24 h. Exposition of these nanostructures to water for longer periods (several weeks) resulted in the formation of large blue crystals of complex **2**. On the contrary, sonication of **1** in ethanol where no chemical conversion is detected, yielded sheet-like 2D flakes. Remarkably, dispersion of such flakes in water leads to the chemical interconversion to complex **2** and the formation of rods, similar to those obtained by direct sonication of complex **1** in water. These novel morphologies tend to aggregate over days in hexagonal-shaped microstructures reminding to the shape of crystals of **2**. These results demonstrate that the combination of a solvent of choice with US open new venues for the obtaining of novel CPs nanostructures with a high chemical/morphological control.

## 4 Materials and methods

### 4.1 Chemicals and materials

Solvents and starting materials were purchased from Scharlau (Scharlab S.L., Barcelona, Spain) and Sigma − Aldrich (Merck, Madrid, Spain). All the materials were used as received, without further purification.

### 4.2 Synthesis of complex {Cu_2_(L)(DMF)_2_}_n_**(1)**

Crystals of CP **1** were obtained by dissolving pyromellitic acid (LH_4_) (101.7 mg, 0.4 mol) and Cu(NO_3_)_2_·2.5H_2_O (24.4 mg, 0.1 mol) in 10 mL of a N,N-dimethylformamide (DMF): Dimethylacetamide (DMA) mixture (1:1) and stirring the solution magnetically in a vial for 30 min at room temperature (RT). Afterwards, the mixing was heated up to 100 °C for 72 h. The synthetic methodology used is schematically depicted in Fig. 2b. Crystals of **1** were then slowly cooled down to RT, cleaned and resuspended in the same solvent used for the synthesis. The cleaning process was performed by repeating centrifugation cycles (13300 rpm, 5 min) at 4 °C. In between cycles, the supernatant was removed and replaced for new DMF:DMA (1:1) solutions. Final crystals were collected and dried for further characterization and sonication. FT-IR (KBr, pellet, cm^−1^): 3435 (m), 3013 (w), 2933 (m), 2813 (w), 1663 (s), 1639 (s), 1527 (w), 1492 (w), 1446 (m), 1442 (w), 1393 (s), 1335 (m), 1284 (w), 1253 (w), 1139 (m), 1108 (m), 1062 (w), 1019 (w), 905 (w), 871 (w), 821 (m), 797 (m), 762 (w), 724 (w), 680 (m), 560 (m), 517 (m). Elemental analysis calculated for [Cu_2_(L)DMF_2_]n: C, 36.72%, H, 3.08%, N, 5.35%. Found: C, 37.20%, H, 3.14%, N, 5.37%.

### 4.3 Synthesis of {[Cu(L)(H_2_O)_3_]·H_2_O}_n_**(2)**

Crystals of CP forming complex **2** were synthesized by immersing clean crystals of **1** in water in a closed vial. After one month, crystals of **1** have regrouped forming hexagonal bluish bigger crystals. Crystals of **2** were isolated and dried at vacuum for single x-ray diffraction (SXRD). FT-IR (KBr, pellet, cm^−1^): 3443 (m), 3263 (m), 3201 (m), 1626 (s), 1583 (s), 1494 (m), 1418 (s), 1371 (s), 1323 (m), 1260 (w), 1186 (w), 1139 (m), 1053 (w), 935 (w), 870 (m), 820 (m), 763 (w), 699 (m), 572 (m), 539 (m), 463 (w), 439 (w).

### 4.4 Ultrasonication

Ultrasonication of crystals of complex **1** was performed by immersing an US microtip into a vial containing a dispersion of crystals of **1** (4.6 mg) (final concentration 1 mg/mL). US were applied to the mentioned dispersions. The equipment used was Branson Digital Sonifier SFX 550 sonicator (Emerson, St. Louis, MO, USA) which has an effective power of 550 W with a tip diameter is 1/8 in.≈0.3175 cm. The total surface then generating the ultrasound is π(0.3175/2)^2^ = 0.0791 cm^2^. Therefore, the power intensity in this case is 550*0.25/0.0791 = 1737 W·cm^−2^. The tip was used with 25% amplitude of its 550 W power and the sonicated volume was the one corresponding to the crystal suspension (4 mL). Therefore, the power density in this case is 550*0.25/0.004 = 34375 W·L^-1^. Crystal dispersions were maintained under magnetic stirring for the homogenization of the sample and kept at RT by a water–ice bath in order to avoid thermal decomposition. US were applied in pulses of 2 min, followed by 30 s of rest between steps at 25% amplitude energy of the tip. The samples were sonicated for 1, 1.5, 2 and 3 h in order to optimize the yield and size of the nanostructures. Aliquots were taken for further analysis. After the sonication process, suspensions of non-exfoliated bulk material coexisted with the nanostructures. Thus, a centrifugation step was performed in order to separate the colloidal suspensions (supernatant) from the non-exfoliated material (pellet). Sorvall Legend Micro 17R centrifuge (Thermo Scientific) was used. Aliquots from the suspensions were centrifuged varying parameters like time, temperature and rpm. After sonication and centrifugation, final colloidal suspensions were kept under agitation in IKA HS 260 basic shaker. Studies of ultrasonication with the US bath were performed with equipment Elma S40H Elmasonic (Elma Schmidbauer GmbH, Singen, Germany). This equipment has an effective power of 140 W. The bath inner dimension in width, depth and high are 240 mm, 137 mm and 150 mm respectively. The total surface generating the ultrasound is 1460 cm^2^. Hence, the power intensity with the bath is 140/1460 = 0.096 W·cm^−2^. The bath, with a power of 140 W, was filled to its maximum which is 4.25L. This corresponds to a power density of 140/4.25 = 32.9 W·L^-1^.Temperature was kept at RT in order to avoid thermal decomposition of the crystals.

### 4.5 Physicochemical characterization

Optical images (OM) from bulk crystals of the complexes were obtained with a Nikon Eclipse LV100 (Nikon Instruments Inc. Melville, NY, USA) microscope. Samples from all complexes were deposited on a glass substrate by drop casting. Morphology was evaluated from this technique.

Scanning Electron Microscopy (SEM) images were collected in order to study morphology as well as a population statistics analysis of the bulk crystals and the nanostructures. The images were obtained in a FEI Quanta 650 FEG SEM (Thermo Fisher Scientific, Eindhoven, The Netherlands) using secondary electrons mode with a beam voltage of 20 kV and a chamber pressure of 10^-5^ Pa. The working distance was set at 10 mm. Samples were prepared by two methods. On the one hand, by drop casting depositing a drop of the colloidal suspensions on aluminium stubs. On the other hand, by spin coating on glass substrates to avoid large aggregation or solvent effects using a WS-650MZ-23NPP Spin Coater (Laurell Technologies Corporation, North Wales, PA, USA). These glass substrates were then deposited onto carbon film stubs. Afterwards, samples were coated with 5 nm thickness of platinum using Leica EM ACE600 (Wetzlar, Germany) metal pulveriser. Cross-section images were taken tilting the sample 70°. Subsequently, SEM images were treated with ImageJ software (NIH, USA) for statistical analysis of the crystal population. Morphology and size of the nanostructures after sonication and isolation were also analysed by SEM images. Images of the suspensions sonicated at different times and with different centrifugation parameters were taken at different amplifications in order to evaluate the optimum sonication time and isolation process.

Infrared spectroscopy (FT-IR) of the bulk crystals was performed for ensuring the chemical stability since infrared absorption promoting one vibrational mode to a higher energy one is characteristic of each material. FT-IR spectra of the crystals were obtained on a Tensor 27 FTIR spectrometer (Bruker Optik, GmbH, Berlin, Germany). Tensor 27 is equipped with a RT detector and a mid-IR source (4000 to 400 cm^−1^). Measurements were made from a KBr matrix containing the crystals. A prior step was needed, which consisted on pressing a mix of KBr and the bulk crystal. A background spectrum was also necessary before sample measurements. On the other hand, FT-IR spectra of the nanostructures were recorded on a Hyperion 2000 FT-IR microscope (Bruker Optik, GmbH, Ettlingen, Germany). Hyperion 2000 in reflection mode is equipped with a nitrogen-cooled mercury-cadmium-telluride (MCT) detector (InfraRed Associates, Inc., Stuart, FL, USA) using a 15x reflection objective, a gold mirror as a reference and scanning for 30 min with a resolution of 4 cm^−1^. Samples were prepared by drop casting of the colloidal suspensions on a gold surface. Subsequently, data collected was treated with Opus software.

SXRD pattern of **1** was resolved by sending good quality crystals to an external service (Institut de Chimie des Substances Naturelles, CNRS, France). The experimental structure was obtained using a RIGAKU XtaLabPro diffractometer equipped with a microfocus sealed tube (Mo λ = 0.71073 Å) generator coupled to a double-bounce confocal Max-Flux® multilayer optic and a HPAD PILATUS3R 200 K detector. While structure of **2** was resolved by sending good quality crystals to another external service (Instituto de Ciencia Molecular, Universitat de València, Spain). A suitable block-shaped crystal of **2** was selected and mounted on a mylar loop support on an SuperNova, Single source at offset, Sapphire3 diffractometer. X-ray data were collected at 120 K. Data were measured using ω scans with MoKα radiation (λ = 0.711 Å). The total number of runs and images was based on the strategy calculation from the program CrysAlisPro (Rigaku, V1.171.38.41q, 2015). The maximum resolution that was achieved was ɵ = 25.027° (ρ = 0.84 Å). A multi-scan absorption correction was performed using CrysAlisPro 1.171.38.41q (Rigaku Oxford Diffraction, 2015) using spherical harmonics as implemented in SCALE3 ABSPACK. The structure was solved in the space group *I*2*/a* (# 15) determined by the XT (Sheldrick, 2015) structure solution program with the Intrinsic Phasing solution method and by using Olex2 (Dolomanov et al., 2009) as the graphical interface. The model was refined with version 2017/1 of XL (Sheldrick, 2008) using Least Squares minimisation. All non-hydrogen atoms were refined anisotropically. Hydrogen atom positions were calculated geometrically and refined using the riding model. All structures were analyzed with Mercury software. Powder X-Ray Diffraction (PXRD) of the bulk crystals were obtained with PANalytical X’Pert PRO MRD (Multipurpose Diffractometer) (Malvern PANalytical, Dusseldorf, Germany) equipped with a CuKα radiation source (λ = 1.54184 Å). Grazing angle X-Ray Diffraction (GXRD) patterns of the nanostructures were obtained by using grazing angle mode with PANalytical X’pet Pro MPD (Materials Research Diffractometer) equipment with a wavelength of CuKα anode (λ = 1.54184 Å) and incident angle of 1.55°. Samples were prepared by drop casting using silicon substrates. Measurement conditions were 2θ range (10 − 45°); step size = 0.03°; counting time/time per step = 14 s.

Dynamic Light Scattering (DLS) measurements were performed using a Zetasizer Nano ZS 3600 (Malvern Instruments, UK). All measurements were done by introducing the dispersions in a glass cuvette for size measurements. Data reported are values coming from the mean of measurements for each sample which were measured per triplicated. The data was collected with the Zetasizer 7.04 software.

## Declaration of Competing Interest

The authors declare that they have no known competing financial interests or personal relationships that could have appeared to influence the work reported in this paper.
