# Supplementary material for: Solvent-tuned ultrasonic synthesis of 2D coordination polymer nanostructures and flakes
Source: Ultrason Sonochem. 2020 Dec 24;72:105425. doi: 10.1016/j.ultsonch.2020.105425 (PMC7803821; doi:10.1016/j.ultsonch.2020.105425)
Supplement: Supplementary data 1 [file mmc1.docx]

# Supporting information

# for

# *Solvent-tuned ultrasonic synthesis of 2D coordination polymer nanostructures and flakes*

# by Pepió et al.

**S1. Characterization of complex** 1

**Table S1**. Crystallographic data from complex **1** structure.

| Crystallographic data | |
| --- | --- |
| Chemical formula | C_8_H_8_CuNO_5_ |
| Molecular weight | 261.54 g/mol |
| Crystal system, space group | Monoclinic, *I*2*/m* |
| Temperature (K) | 293(2) |
| a, b, c (Å) | 6.2197(6), 8.6533(7), 18.172(3) |
| α, β, γ (°) | 90, 92.711(11), 90 |
| V (Å^3^) | 976.94(19) |
| Z | 4 |
| Radiation type | MoKα 0.70173 |
| µ (mm^-1^) | 2.235 |
| Crystal size (mm) | 0.03x0.02x0.02 |
| Tmin, Tmax | 0.87928, 1.00000 |
| No. of measured, independent and  observed [I > 2σ(I)] reflections | 4228, 1102, 959 |
| R_int_ | 0.0491 |
| Refinement | |
| R[F2 > 2σ(F2)], wR(F2), S | 0.0372, 0.0858, 0.0830 |
| No. of reflections | 1100 |
| No. of parameters | 87 |
| H-atom treatment | H-atom parameters constrained |
| Δ>max, Δ>min (e Å^-3^) | 0.525, -0.654 |

**Table S2.** List of bonds and angles for complex **1**

| Bond | Length (Å) | Bond | Length (Å) |
| --- | --- | --- | --- |
| C1-O1 | 1.26(3) | C3-H3A | 0.96 |
| C1-C2 | 1.51(1) | C3-H3B | 0.96 |
| C1-O2 | 1.233(9) | C3-H3C | 0.96 |
| N1-C3 | 1.45 | O3-C5 | 1.26 |
| N1-C5 | 1.3 | C4-H4 | 0.93 |
| N1-C6 | 1.44 | C5-H5 | 0.93 |
| O1-Cu1 | 1.99 | C6-H6A | 0.96 |
| Cu1-O3 | 2.121 | C6-H6B | 0.96 |
| C2-C4 | 1.391 | C6-H6C | 0.96 |

**Figure S1. (**a) Comparison of the PXRD pattern of a bulk synthesis of complex **1** with that simulated from the single crystal data using the *Mercury* software, (b) FT-IR spectra of complex **1** and the free ligand pyromellitic acid, for comparison purposes and (c) EDX analysis of **1**.


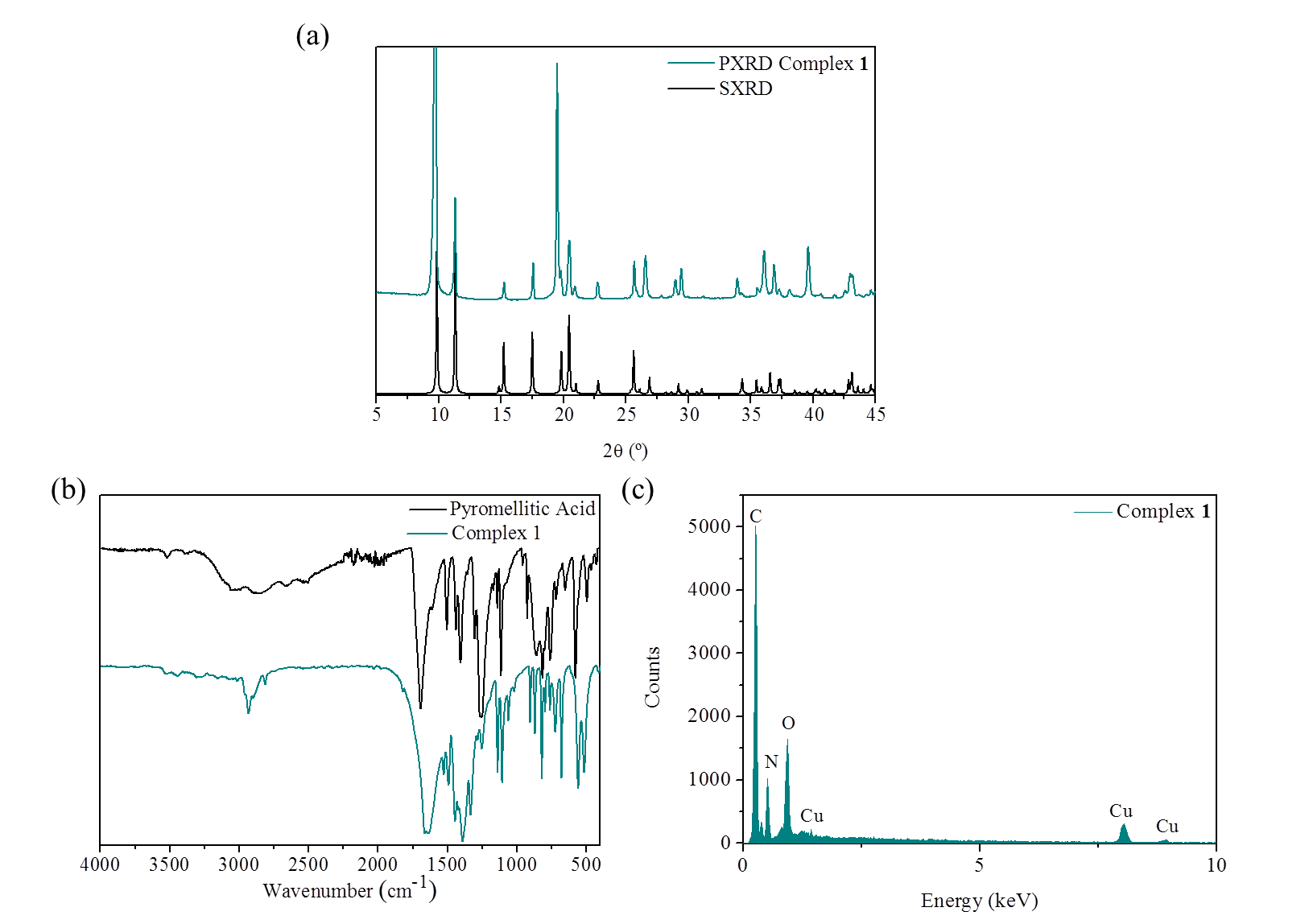


Figure S1 compares the PXRD data of the as-synthetized crystals with the simulated one form SXRD with Mercury software. Both spectra were in good agreement and small difference in some peaks could be attributed to the preferential directions found in experimental PXRD. These results confirmed the reproducible and robust synthesis of **1**. The broad FT-IR peak present in pyromellitic acid centred at 2800 cm^-1^ showed that the stretching of COO-H bond was replaced by a multiplet signal that represents the C-H stretching present in alkanes. This peak could indicate the presence of solvent (DMF) within the structure, intercalated between bands (or due to a not completed drying of the solvent). Also, strong peak around 1650 cm^-1^ of C=O stretching from carboxylic acids was present and displaced at lower wavenumber in the bulk 2D-CP as usually observed after the coordination of the ligand with the metal. Obtained results of EDX analysis confirmed the presence of copper within the structure and the other elements expected such as N coming from nitrate counter ions and possibly from DMF solvent as its presence was seen in FT-IR analysis.

**S2. Irreversibility of water changes in complex** 1

**Figure S2**. (a) FT-IR spectra and (b) PXRD data of crystals of **1** immersed in water for 24 h and afterwards back in DMF:DMA for additional 24 h and two months. Water-induced changes were irreversible mostly due to the chemical transformation involved in the process.

**S3. Evolution of the complex** 2 **formation**

**Figure S3.** Time-dependence PXRD evolution of crystals of complex **1** in contact with water.


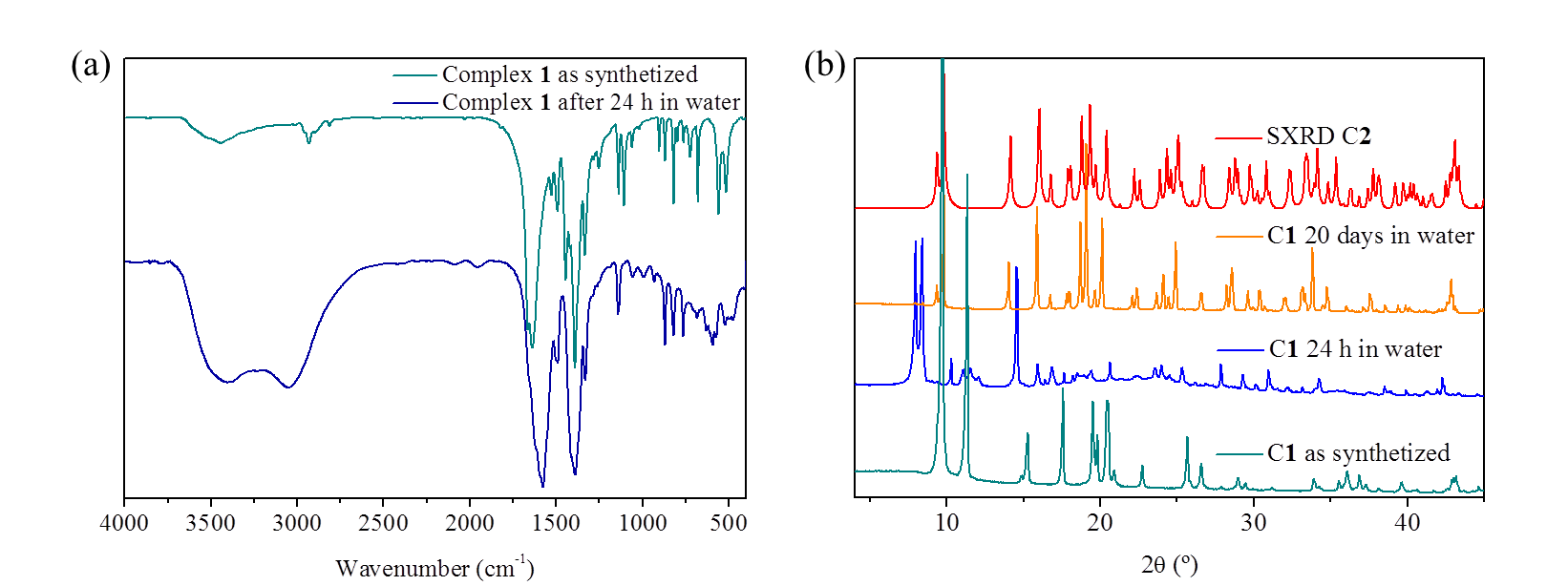


**S4. Characterization of complex** 2

Chemical characterization on **2** was performed by means of FT-IR and EDX analysis. Regarding FT-IR spectra in Figure S4a it could be noticed the presence of (COO)^-^ groups of the ligand within the ranges of 1610-1550 cm^-1^ (asymmetric stretching) and 1550-1450 cm^-1^ (symmetric stretching). The mentioned peaks appeared displaced with respect the ones present in the ligand due to the coordination with the metal. Additionally, it could be observed O-H stretching signal as a broad peak in the range of 3600-2700 cm^-1^ corroborating the presence of water molecules as seen in SXRD analysis. The transition of **1** into **2** did not show chemical decomposition, as intuited after 24 h. Likewise, EDX (Figure S4b) confirmed the expected elements in the complex. For instance, N atoms did not appear, indicating that DMF molecules have been completely substituted.


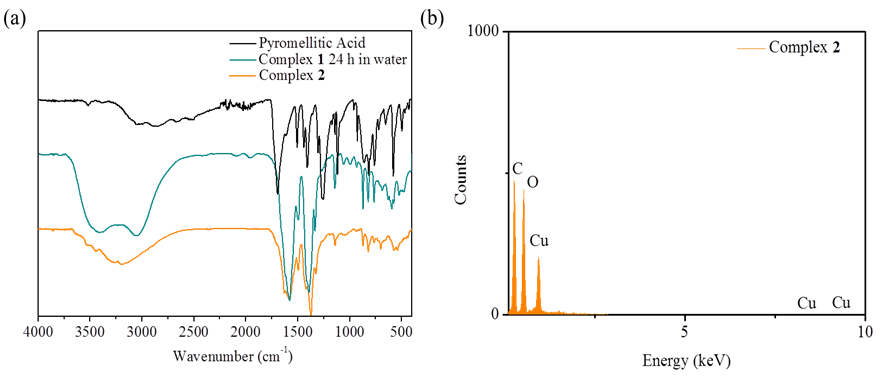


**Figure S4.** Chemical characterization of **2**: (a) FT-IR spectrum and (b) EDX analysis.

**
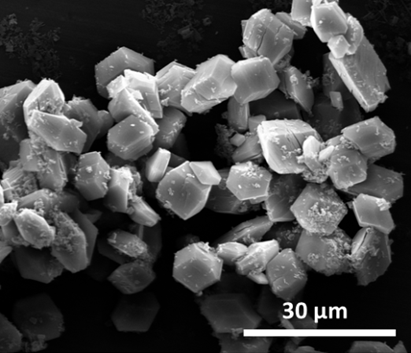
**

**Figure S5.** SEM image of crystals of complex **2** obtained by dispersion of crystals of complex **1** in water for several weeks.

**S5. Sonication of complex** 1 **in water**

***Tip sonication*.**

**Table S3**. Tip sonication parameters used for the exfoliation of complex **1** in water. Dynamic light scattering (DLS) values correspond to the mean of three measurement runs in a sample indicating hydrodynamic diameter. Tim amplitude (%): 25 in all the cases. PDI: polydispersity index.

***Bath sonication*.** US bath exfoliations were tested in the two representative solvents, water that modifies the structure and ethanol, which did not as previously described. Dynamic light scattering (DLS) results are shown in Table S4 and Figure S6. It could be observed that the polydispersity index (PDI) values were relatively monodispersed, especially at longer sonication periods, as US were homogeneously applied all over the dispersion.

**Table S4.** Exfoliation parameters of the delamination of **1** in different solvents with an US bath. DLS size correspond to the mean of three measurement runs in a sample indicating hydrodynamic diameter.

| Solvent | Time (h) | Size (nm) | PDI |
| --- | --- | --- | --- |
| Water | 2 | 87.4 ± 8.5 | 0.486 ± 0.052 |
| EtOH | 2 | 141.4 ± 6.9 | 0.232 ± 0.001 |
| Water | 6 | 79.4 ± 7.3 | 0.195 ± 0.019 |
| EtOH | 6 | 168.1 ± 57.9 | 0.217 ± 0.017 |


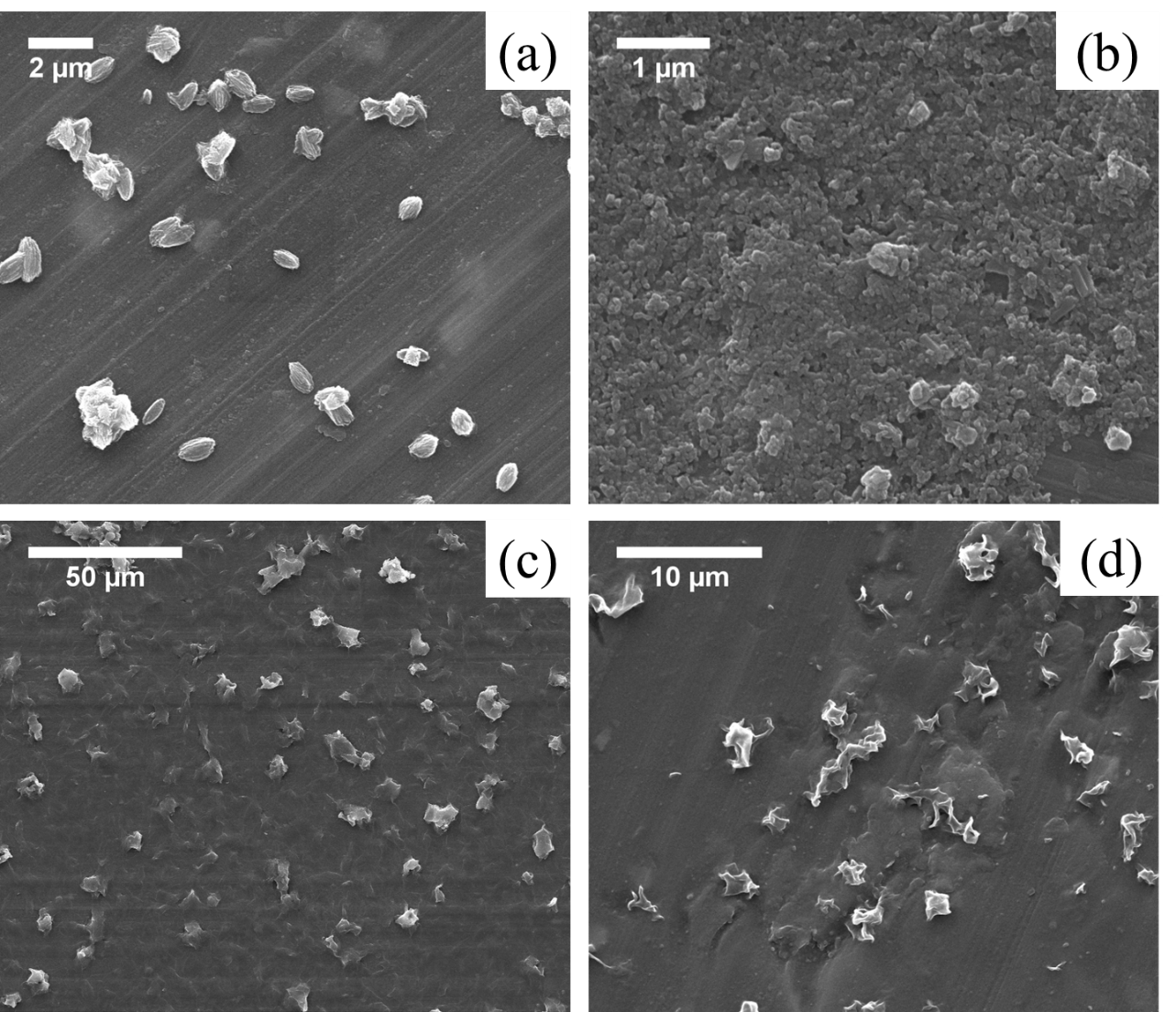


**Figure S6**. SEM images of 2D-CP exfoliated with the US bath in water for (a) 2 h and (b) 6 h. SEM images of 2D-CP exfoliated with the US bath in EtOH for (c) 2 h and (d) 6 h.

**S6. Optimization of the centrifugation process**


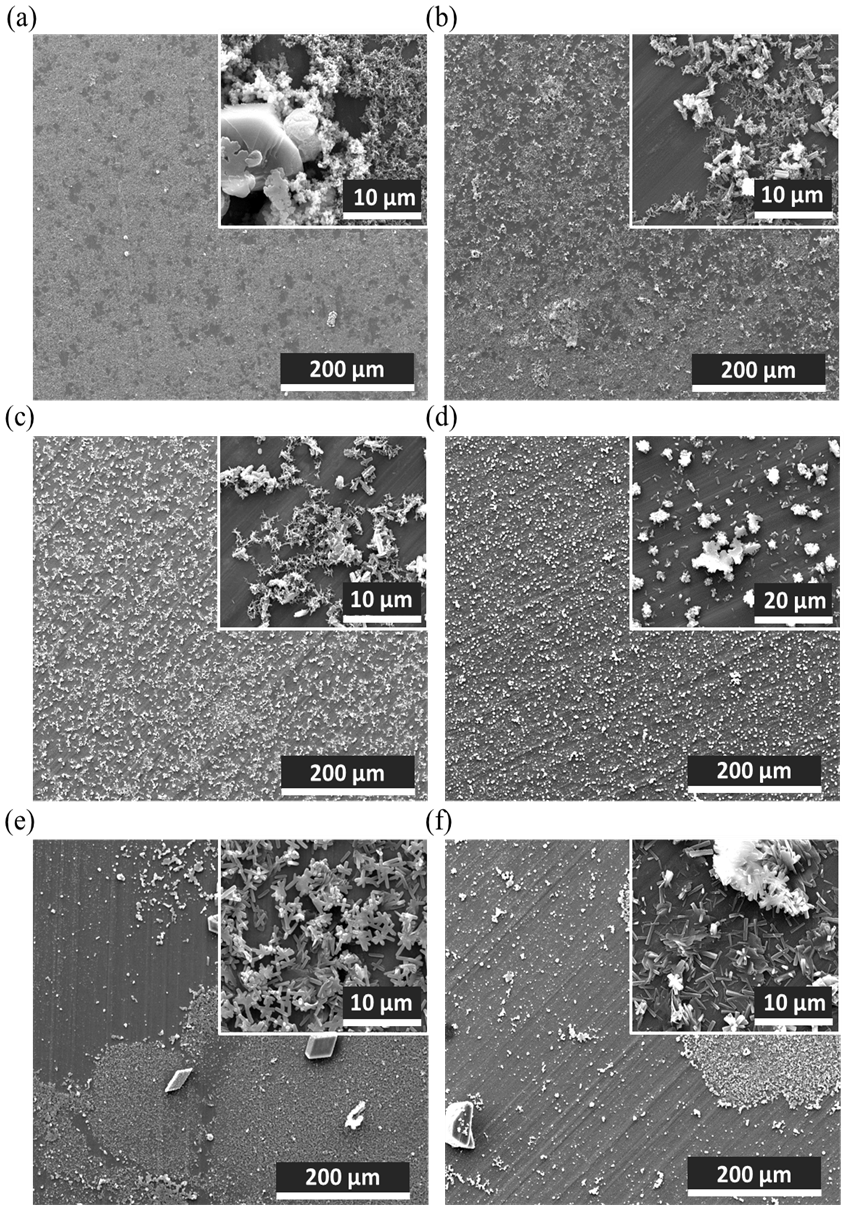


**Figure S7.** Centrifuge optimization was performed in order to isolate the nanostructures from the bulk material at a constant temperature of 4 ºC, 13300 rpm and different times. SEM images of the colloidal suspensions sonicated for 2 h and centrifuged at 13300 rpm and 4 ºC for (a) 2 min, (b) 5 min, (c) 7 min, (d) 10 min, (e) 15 min and (f) 20 min. It can be observed that bulk material was removed almost completely in all the cases though at 2 min the size distribution was still broad and in less degree at 5 and 7 minutes. On the other side, at 15 and 20 minutes samples showed homogeneous morphology. However, nanostructures were larger, indicating a possible effect of long centrifugation times on the morphology. Thus, 10 minutes was selected as the optimum time since it presented the more homogeneous material and narrower size distribution.

**S7. Characterization of nanostructures**


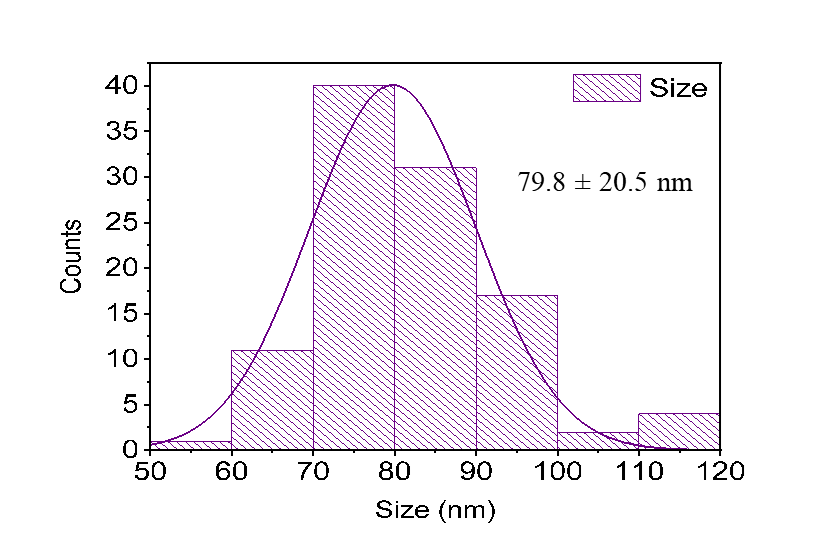


**Figure S8.** Size distribution of the nanospheres obtained when sonicating **1** in water


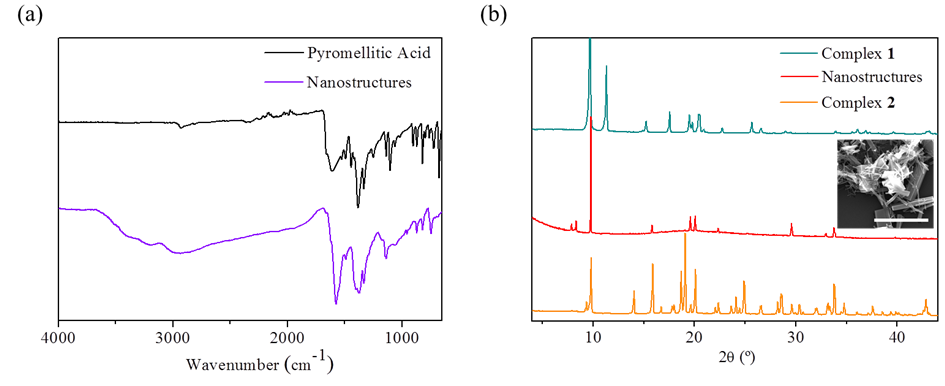


**Figure S9.** (a) FT-IR of the final nanorods material compared with the ligand and (b) GXRD patterns of the final nanorods material compared with PXRD of complex **1** and **2**. The sample with which GXRD was measured was also analyzed by SEM and is showed in the figure where the scale is 5 µm.


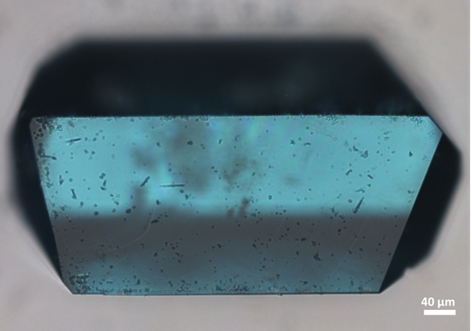


**Figure S10**. Crystal obtained after 3 months from the sonication of crystals of complex **1** in water.


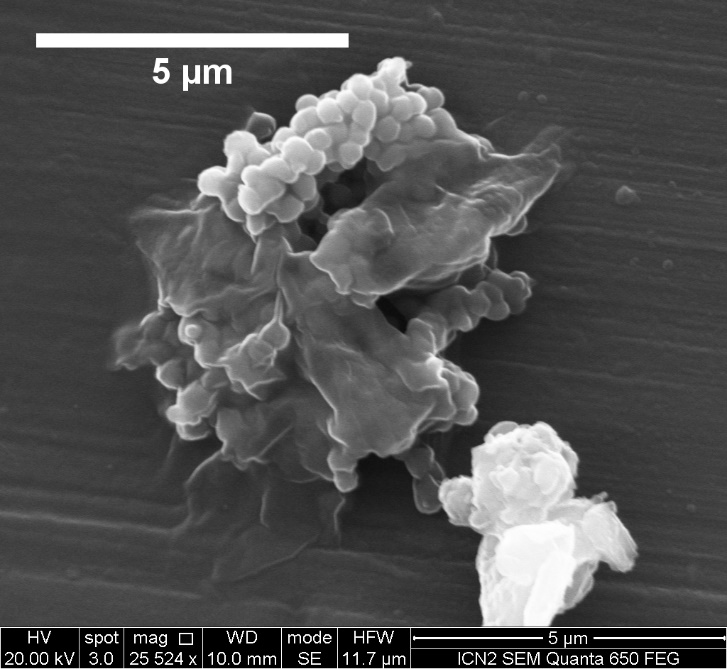


**Figure S11**. SEM image of flakes exfoliated in EtOH and resuspended in water previous to EtOH evaporation
